# Midregion PTHrP and Human Breast Cancer Cells

**DOI:** 10.1100/tsw.2010.97

**Published:** 2010-06-01

**Authors:** Claudio Luparello

**Affiliations:** Dipartimento di Biologia Cellulare e Dello Sviluppo, Università di Palermo, Italy

**Keywords:** breast cancer cells, MDA-MB231, midregion PTHrP, PTHrP (38-94), gene expression, DNA status, apoptosis, protein degradation, nuclear import

## Abstract

PTHrP is a polyhormone undergoing proteolytic processing into smaller bioactive forms, comprising an N-terminal peptide, which is the mediator of the “classical” PTH-like effect, as well as midregion and C-terminal peptides. The midregion PTHrP domain (38-94)-amide was found to restrain growth and invasion *in vitro* of some breast cancer cell lines, causing striking toxicity and accelerating death; the most responsive being MDA-MB231, whose tumorigenesis was also attenuated *in vivo*. In addition, midregion PTHrP appears to be imported in the nucleoplasm of cultured MDA-MB231 cells and *in vitro*, it can bind chromatin of metaphase spread preparations and also an isolated 20-mer oligonucleotide, thereby appearing endowed with a putative transcription factor–like DNA-binding ability. The object of this review is to discuss collectively and critically both precedent and more updated data obtained in the lab, the latter arising from assays on DNA status, and gene and protein expression patterns of treated cells, aiming to check whether the cytotoxicity of the peptide may result from a reprogramming of gene expression towards apoptotic death or, instead, it is to be ascribed to an unprogrammed perturbation of cell functions.

## BREAST CANCER AND PTHrP

The human breast is a modified sweat gland that originates from the epidermal mammary ridge, whose epithelial component in the postpubertal age consists of a monolayer of polarized luminal cells organized into branching ducts terminating in multiple acini. The epithelial and myoepithelial component of the gland, leaning onto the basal lamina, is embedded in an abundant interstitial stroma composed of fat tissue, fibrous and loose connective tissue, and blood vessels. It is generally acknowledged that cancer development by the breast luminal epithelium, apart from multistep genetic alterations, is strongly influenced by the network of interactions occurring between cancer cells and the novel microenvironment surrounding them. Within the original tissue structure, in fact, newly synthesized soluble factors, providing additional and different biological stimuli, and macromolecular polymers of the stromal matrix, exposed after the dissolution of the basal lamina, become available for epithelial cell attachment and signalization[e.g., 1,2,3,4].

Substantial evidence indicates that one of those microenvironmental factors strongly involved in breast tumor progression is parathyroid hormone–related protein (PTHrP), whose involvement in ductal branching morphogenesis of the developing breast and in the physiology of lactation has been widely documented[5,6,7,8,9,10]. PTHrP, classically regarded as the mediator of the humoral hypercalcemia of malignancy syndrome, is the product of a gene spanning more than 15 kb of genomic DNA and exhibiting a complex organization in humans, where it generates multiple mRNA variants through alternative splicing events and utilization of different transcriptional start sites. The initial translation products are three isoforms of either 139, 141, or 173 amino acids with distinct C-terminals, displaying sequence homology with PTH at the extreme N-terminus, which allows the binding to the same G protein–linked receptor PTH1R[11, for a review]. PTHrP is a polyhormone undergoing a proteolytic processing, possibly tissue-specific, by members of the subtilisin family of proteases (e.g., furin, PC1/3, PC2, PACE4, PC8; see [12] and references therein) into smaller bioactive forms, comprising an N-terminal peptide, which is the mediator of the "classical" PTH-like effect, as well as midregion and C-terminal peptides. It must be remembered that one of the original tumors from which PTHrP was purified and sequenced, because of the elevated concentration of its circulating form, was breast cancer[13,14]. Moreover, it is now well known that PTHrP is involved centrally in both the osteotropism of breast cancer and in the onset of the tumor-associated humoral hypercalcemic syndrome resulting from systemic overproduction of the protein and its local release at skeletal metastasis sites with the following activation of bone resorption[12,15]. Indeed, immunohistochemical and *in situ* hybridization studies indicated that *PTHrP* expression was higher and more frequent in breast tumor skeletal metastases than in the parental primary tumor[16,17,18]. Auto- and intracrine regulation of breast tumor cells by PTHrP has been supported by experimental data in both *in vivo* and *in vitro* model systems[e.g., 19,20,21,22,23,24]; in addition, breast stromal cells, both embryonic and neoplastic, and myoepithelial cells have also been proven to synthesize and secrete PTHrP, thus critically contributing to the maintenance of epithelial/mesenchymal signaling paracrine circuits in mammary tissue[25,26,27].

An aspect of PTHrP that attracted the attention of several laboratories, including ours, is that its biological effects actually exerted the discrete fragments originating from its post-translational processing, which can also mediate opposing functions and whose properties have been investigated in different *in vitro* model system[28, for review]. Within this context, concerning breast cancer, we have previously demonstrated that (1) *PTHrP* expression was restricted to subpopulations of the heterogeneous 8701-BC cell line, derived from a biopsy fragment of a primary neoplasm, endowed with a more "aggressive" phenotype[29], (2) its expression in parental 8701-BC cells could be drastically modulated by modifications of the *in vitro* microenvironment, such as cell seeding onto collagen films, or addition of growth factors, hormones, or chemicals to the media[30,31], and (3) (1-34), (67-86), and (107-138), but not (38-64), PTHrP domains actively controlled the proliferative and invasive behavior as well as the expression of genes encoding for stress response proteins and extracellular lytic enzymes in both the parental cell line and selected clonal lines[32,33,34].

Here, the findings obtained to date about the cytotoxic activity exerted on breast cancer cells by PTHrP (38-94)-amide, a fragment that has potentially important therapeutic implications for antineoplastic molecular modeling, will be discussed.

## PTHrP (38-94), THE AUTHENTIC MIDREGION PHYSIOLOGIC SECRETORY FORM, IS BIOLOGICALLY ACTIVE ON BREAST TUMOR CELLS

Although a number of literature reports demonstrated the biological properties of different midregion PTHrP domains, such as (38-64) and (67-86), the collective data of Soifer et al.[35], Yang et al.[36], and Wu et al.[37] established unequivocally that the actual midregion physiologic secretory form of the polyhormone was PTHrP (38-94)-amide. The first experimental data on the effect exerted by this specific fragment were enclosed in the paper by Wu and coworkers[37], and were related to its ability to stimulate transients in cytosolic calcium in rat insulinoma RIN and human cervix carcinoma YCC cultured cells, and transplacental calcium transport *in vivo*. Successive studies performed by Strid et al.[38] on PTHrP (38-94) and $Ca^{++}$ fluxes demonstrated that the peptide was able to stimulate selectively the ATP-dependent transport of the ion by syncytiotrophoblast basal membrane vesicles through the $IP_3$-DAG-PKC second messenger pathway. In 2001, a paper was published that explored the effect of PTHrP (38-94)-amide on viability, proliferation, and invasiveness of six different breast cancer cell lines (Hs578T, MDA-MB231, T47-D, 8701-BC, and its clonal lines BC-3A and BC-61), representing the spectrum of breast tumors encountered in medical practice and spanning the range of histochemical subtypes, estrogen receptor status, vimentin expression, activity in chemotaxis and chemoinvasion assays, and invasive behavior in nude mice. It demonstrated that, different from the other PTHrP fragments tested, i.e., (1-34), (38-64), (67-86), and (107-138)[32,33,34], PTHrP (38-94)-amide, administered at 1-nM concentration, was able to restrain growth and invasion *in vitro*, as well as to cause striking toxicity and accelerate death of the panel of cells assayed; the most responsive being MDA-MB231, whose tumorigenesis in immunocompromised mice was further studied and appeared to be restrained markedly by daily intratumoral inoculation of the peptide[39].

These results prompted a more detailed study on tumor cell-PTHrP interactions and the molecular pathway(s) through which this midregion peptide may accomplish its powerful effects on the phenotypic modulation of mammary carcinoma cells.

## PTHrP (38-94) IS A PUTATIVE TRANSCRIPTION FACTOR–LIKE MOLECULE

It is known that PTHrP contains a nuclear localization sequence (NLS) mediating importin β–driven nuclear import, and that nucleocytoplasmatic shuttling of the protein is cell cycle dependent[e.g., 40,41]; PTHrP NLS has been localized in the sequence spanning amino acids 87 to 106, with the preceding sequence, i.e., (71-82), likely required to guarantee a more stable importin binding. Being an NLS-possessing peptide, midregion PTHrP, once it has gained access to the nucleoplasm, could play potential transcription factor–like roles. As a support to this hypothesis, the intracrine role for PTHrP in cell growth, adhesion, migration, invasion, and integrin expression regulation has been documented for MCF-7 breast tumor cells overexpressing the wild-type vs. NLS-mutated protein[22,42]. Mobilization of intracellular calcium has also been observed in different breast cancer cell lines treated with PTHrP (67-101), whose ability to translocate to the nucleus was reported by Kumari et al.[43]. In addition, intracrine effects of midregion PTHrP domains have been described in other experimental models[e.g., 44,45,46].

One of the questions that remained unsolved was whether the interaction between PTHrP and nuclear chromatin would be direct, or if other nuclear components participating in the formation of putative supramolecular regulatory complexes would be necessary. To this purpose, living breast cancer MDA-MB231 cells were treated with midregion PTHrP, demonstrating its accumulation in the cell nucleoplasm at interphase[47]. Moreover, although lacking *in vivo* counterparts so far, *in vitro* results were obtained that confirmed the direct chromatin binding by PTHrP (38-94)-amide, which was shown to "decorate" chromosomes of metaphase spread preparations from MDA-MB231 cells in a selective and stable manner, with a conspicuous number of evenly distributed, discrete and intense, fluorescent spots that display no accumulation at specific chromosomal sites. Interestingly, the "decoration" pattern appears to be cell type specific since, when preparations from immortalized, nonmalignant, mammary epithelial cells were used, a different number and localization of hybridization signals per genome was observed (Sirchia, Caradonna and Luparello, unpublished data). In the paper by Sirchia et al.[47], a DNA sequence recognized by the protein, i.e., 5'-GAGTAGAATTCTAATATCTC-3, was also identified; this sequence displayed identity with a number of human genomic DNA sequences, two of them located in chromosomes 8q23 and 21q22.3, as well as with a segment of mitochondrial DNA encoding for an rRNA. The specific requirements for optimal binding were also assessed, employing a combination of "whole genome"/conventional PCR, EMSA, and DNAse foot-printing techniques. In particular, the collective data indicated that the binding was selective, required double-stranded DNA, and was effectively competed by preincubation of midregion PTHrP with the anti-PTHrP (34-53) antibody. In addition, the binding was undetectable when either PTHrP (38-64)-amide or (67-86)-amide were submitted to EMSAs in the place of the intact (38-94)-amide fragment, and DNA recognition by PTHrP critically needed the presence of the GA/GAG terminal motifs, albeit repetitive GA and GAG modules were insufficient to create binding sites, and therefore were in some way sequence context dependent. Interestingly, literature data also support the concept that this sequence is involved in protein recognition; in fact, PATCH software analysis (http://www.gene-regulation.com/cgi-bin/pub/programs/patch/bin/patch.cgi) indicated that portions of this 20-mer oligonucleotide were targets for transcriptional modulators of various species, including human GATA-1 and POU2F1. On the other hand, the existence of factors able to bind the GA-rich sequences present in several gene promoters in a context-dependent way, i.e., under the selective control of local sequence characteristics, has been documented in various model systems[e.g., 48].

In light of observations that strongly suggest some nuclear activity for midregion PTHrP endowed with a likely transcription factor–like DNA-binding ability, DNA status, and gene and protein expression patterns in treated cells, were analyzed in order to check whether the cytotoxic activity of the peptide could be consequent to gene expression reprogramming towards apoptotic death or, instead, to an unprogrammed perturbation of cell physiology.

## MIDREGION PTHrP-TREATED MDA-MB231 BREAST CANCER CELLS DISPLAY A GENERALIZED PATTERN OF DNA FRAGMENTATION

First, the plan was to employ a combination of electrophoretic and cytochemical assays to examine whether MDA-MB231 cells treated with 1-nM PTHrP (38-94)-amide, as reported by Luparello et al.[38], displayed oligonucleosomal DNA fragmentation, a generally acknowledged hallmark of apoptosis. The electrophoretic test was accomplished by extracting DNA from lysates of control and treated cells, and submitting the preparations to voltage gradient gel electophoresis (VGGE), as reported by Luparello et al.[49]. The cytochemical assay performed on control and treated cell cultures employed the ApopTag® Peroxidase In Situ Oligo Ligation kit (Serologicals Co.; Norcross, GA) as reported by Luparello and Sirchia[50]. This assay is based on *in situ* ligation in the presence of either biotinylated oligo A, which contains a 3’-dA overhang, or biotinylated oligo B, which is blunt ended; the first oligo being more selective for the detection of “classical” apoptosis than other types of cell death. Successful ligation is put in evidence by precipitation of diaminobenzidine substrate after reaction with streptavidin-horseradish peroxidise.

As shown in Fig. 1, the DNA obtained from cells treated for 6 and 24 h appeared to be in an intact high-molecular-weight form, whereas the DNA extracted after 48 h of incubation with PTHrP displayed an electrophoretic smeared pattern, allegedly ascribable to the occurrence of random degradation events, characteristic of “nonapoptotic” death response (at least in a “conventional” conception). No “ladder-like” pattern of DNA fragmentation was evident in all the experiments performed. A similar result was obtained by cytochemical analysis. In fact, using the apoptosis-discriminating ApopTag technique, more cells stained positively using the “necrosis-related” oligo B vs. the “apoptosis-related” oligo A (Fig. 2).

Thus, this first cumulative data suggested that the PTHrP (38-94)–driven cytotoxic effect on MDA-MB231 breast cancer cells could not be attributed to activation of mechanisms of “classical” programmed cell death. On the other hand, comparison analysis of the PTHrP-binding DNA sequence, cited in the preceding paragraph, with nonhuman DNA sequences deposited in online data banks showed that this sequence was also contained in a 183-bp DNA fragment from *Mus musculus* (acc. nr. AJ403155), which is recognized by vimentin through specific Y and/or F amino acid residues of its N-terminal head domain. In light of this binding ability, vimentin is supposed to participate in the control of both chromatin architecture and DNA recombination/repair events[51,52]. In consideration that the interaction of vimentin with DNA elicits helix-destabilizing configurational changes that render the zone more accessible to the action of T7 endonuclease I[53], and in light of the data on extensive chromatin cleavage following cell incubation with midregion PTHrP, a fascinating hypothesis, still to be validated, could consider the PTHrP-binding sites detected on the different chromosomes as hypothetical selective starting points for chromatin degradation by some still-unidentified endonuclease.

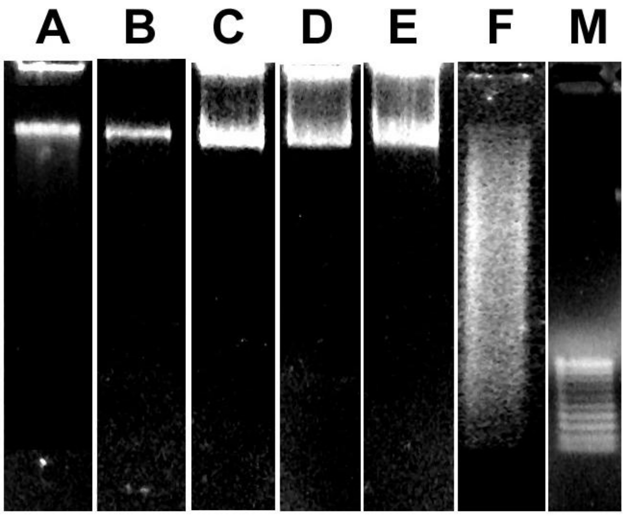

**FIGURE 1.** Representative VGGE of the DNA isolated from MDA-MB231 cells, cultured in control conditions (A,C,E) and in the presence of 1-nM PTHrP (38-94)-amide (B,D,F) for 6 (A,B), 24 (C,D), and 48 h (E,F). M = size marker (100-bp ladder).

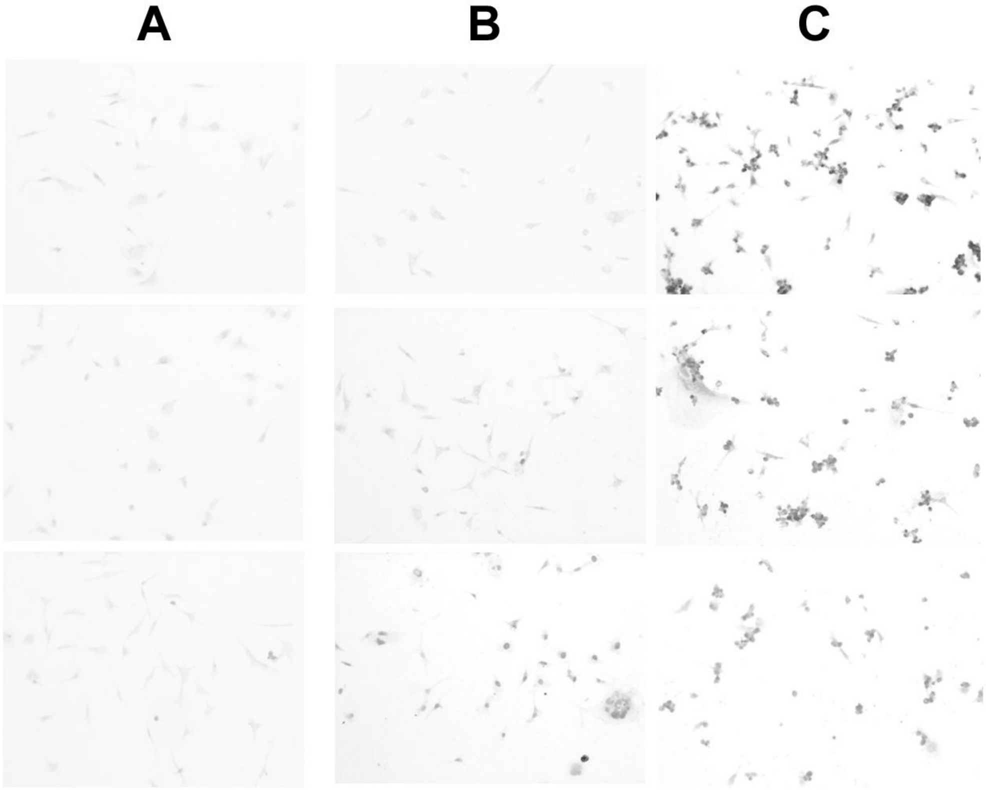

**FIGURE 2.** Panel of micrographs showing ApopTag labeling of MDA-MB231 cells cultured in control conditions (A) and in the presence of 1-nM PTHrP (38-94)-amide (B,C), using oligo A (one-base dT overhang-end detection; B) and oligo B (blunt-end detection; C). Massive positive reaction was obtained only in C. Microscopic magnification: 20×.

## MIDREGION PTHrP-TREATED BREAST CANCER CELLS DISPLAY SELECTIVE CHANGES IN GENE EXPRESSION LEVELS

Whether midregion PTHrP could actively control gene expression was then examined. In light of the results indicating the 20-mer sequence recognized by midregion PTHrP *in vitro*[47], in a first set of experiments, attention was on the genomic sites where it was localized, and appropriately selected the genes for evaluation of their expression levels in control and treated cell samples. In particular, *PCP4/PEP19* was tested, since the sequence is located between two of its exons in the 21q22.3 cytogenetic band, and other genes involved in the control of cell life/death and carcinogenesis, mapping into 8q23 cytogenetic band (*COX6C*, *OXR1*, *FZD6*, *RRM2B*, *EIF3S6*, *EBAG9*); in addition, *HOX7/MSX1* and *WT1*, whose promoters are rich in GA sequences, due to the documented midregion PTHrP affinity to GAG and GA motifs, were also included in the experiments. To further supplement the catalogue of midregion PTHrP-dependent genes, the technique of differential display (DD)-PCR was also applied to cDNA samples obtained from enriched mRNA preparations of both control and treated MDA-MB231 cells[54].

Through a combination of conventional and semiquantitative multiplex (SM)-PCR techniques, the midregion PTHrP-dependent down-regulation of four genes was demonstrated, i.e., *OXR1* (i.e., *Oxidation Resistance 1*); *FZD6* (i.e., *Frizzled Homologue 6*); *COX6C* (i.e., *Cytochrome c Oxidase, subunit VIc*), implicated in cell survival, mitochondrial respiration, and protection from injury; and *HOX7/MSX1* (i.e., *Homeobox 7/Muscle Segment Homeobox, Drosophila, Homolog 1*), a homeotic gene involved in the control of complex intracellular networks. The decreased transcriptional activity of such genes might be potentially involved in PTHrP-mediated cytotoxic effect; on the other hand, although this result could be of further support to the hypothesis of the intrinsic transcriptional modulation capability of the PTHrP fragment, evidence that gene down-regulation is a result of the actual peptide binding to DNA is still lacking. Moreover, DD-PCR experiments identified other genes whose expression levels were affected by incubation with PTHrP (38-94)-amide and whose biological implications in the model system under study are still to be determined. In particular, *A4 differentiation-dependent protein*, also termed *PLP2*, was found to be up-regulated, whereas *TMCO4* was underexpressed[54].

To expand the list of midregion PTHrP-dependent genes in MDA-MB231 cells, we tested whether modifications in the expression levels of genes coding for stress response proteins, and for factors and enzymes involved in the onset of apoptosis, could also occur following incubation of cells with the peptide

Concerning stress response proteins, the data obtained are published herewith for the first time. The panel in Fig. 3, representative of three independent experiments, shows that in conventional PCR assays, a positive signal was found for all the cDNA tested and obtained from cell samples with or without exposition to PTHrP (38-94), indicating that the selected gene expressions are switched on in both experimental conditions. The cDNA preparations were then submitted to triplicate SM-PCR to compare the expression levels of the selected genes. As shown in the panels in Fig. 4, it was found that incubation of MDA-MB231 cells with PTHrP (38-94)-amide was able to modulate the expression levels of only *hsc70* (+3.9 folds), *hsp70* (+2 folds), and *hsp90α* (-2.6 folds) among the stress protein genes. All the standard errors of the mean (s.e.m) were less than 0.05. The up-regulation of *hsp70* and *hsc70* may be interpreted as a typical stress response. Ciocca et al.[55] produced evidence that *hsp70* overexpression in MDA-MB231 cells is associated with acquisition of resistance to the anticancer drug doxorubicin; it is also worth mentioning that Grzesiak et al.[56] recently reported interaction between various PTHrP fragments, including midregion (37-86), and hsp70 expressed on the surface of cancer cells, thereby being somehow involved in the internalization of extracellular PTHrP. On the other hand, up-regulation of *hsc70* (i.e., cognate of hsp70), encoding for another cytosolic molecular chaperone involved in the folding of newly synthesized polypeptides, has been correlated to apoptosis resistance in ovarian cancer cells[57]. Of note, hsc70 is reported to be involved in the so-called “chaperone-mediated autophagy”, in that it stimulates an intracellular pathway of proteolysis that is selective for particular cytosolic proteins that are bound and targeted to the lysosomes for destruction[e.g., 58]; thus, up-regulation of *hsc70* could be seemingly related to the enhanced protein degradation identified by electrophoretic analysis in preparations from PTHrP (38-94)–treated breast cancer cells, as described elsewhere. Moreover, *hsp90α*

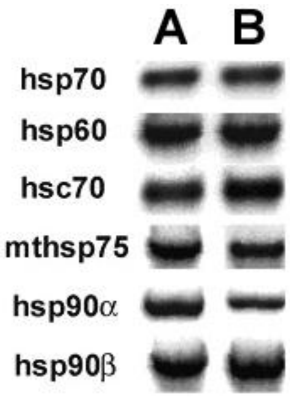

**FIGURE 3.** PCR analysis of expression of genes coding for stress response proteins by control (A) and midregion PTHrP-treated (B) MDA-MB231 cells.

expression level also appears to be correlated to cell growth behavior, since its decrease has been reported to determine the switching off of proliferation in osteoblasts[59], while its increase seems to be involved in the enhanced proliferation of pancreatic carcinomas[60].

Concerning apoptotic factors and enzymes, as reported by Luparello et al.[61], PTHrP appears capable to modulate the expression of *Bcl-xS* (+2.2 folds), *Bad* (+2.7 folds), and, more prominently, *Rip-1* (+4.26 folds), and to switch on the expression of *caspase-2*, *-5*, *-6*, *-7,* and *-8* in MDA-MB231 breast cancer cells. Although the data obtained put in evidence a certain stability of the expression levels for most of the genes coding for apoptosis modulators, nonetheless, the cytotoxic effect triggered by midregion PTHrP on MDA-MB231 could see the involvement of some proapoptotic factors, such as Bad and Bcl-xS, whose up-regulation were found to promote cell death in different breast cancer cell lines, including MDA-MB231[50,62,63,64,65]. According to Tudor et al.[66], alteration of *Bad* transcription or mRNA stability is an early cellular response to stress or drug-induced injury, and a potentially critical regulation point of downstream steps, susceptible to restraint by survival mechanisms that cumulatively govern the ultimate predisposition to apoptosis.

Particularly interesting is the observation that *Rip-1*, a gene encoding for a member of receptor-interacting protein (Rip) family kinases that function as integrators of extracellular and intracellular stresses and crucial regulators of cell survival[67], is up-regulated. *Rip-1* is the gene undergoing the most significant change of expression level among those tested, and its protein product has been reported to trigger prosurvival responses as well as opposite death-inducing mechanisms. Antisense oligonucleotide-mediated down-regulation of *Rip-1* in PTHrP-treated MDA-MB231 cells, although unable to modify cell proliferative behavior, determined the up-regulation of all *caspase* genes tested due, at least in part, to c-Jun-N-terminal kinase (JNK) inactivation[61]. This represents a new example of factors involved in the transcriptional regulation of the apoptotic enzymes, whose molecular aspects are still to be elucidated.

## MIDREGION PTHrP-TREATED MDA-MB231 BREAST CANCER CELLS UNDERGO POTENTIAL MASSIVE PROTEIN DEGRADATION

In a last set of experiments, still at the preliminary stage, the global electrophoretic protein pattern of preparations from control and PTHrP-treated MDA-MB231 cell cultures was checked. To this purpose, cells were directly lysed in flasks and lysates submitted to protein extraction according to Wang et al.[68];

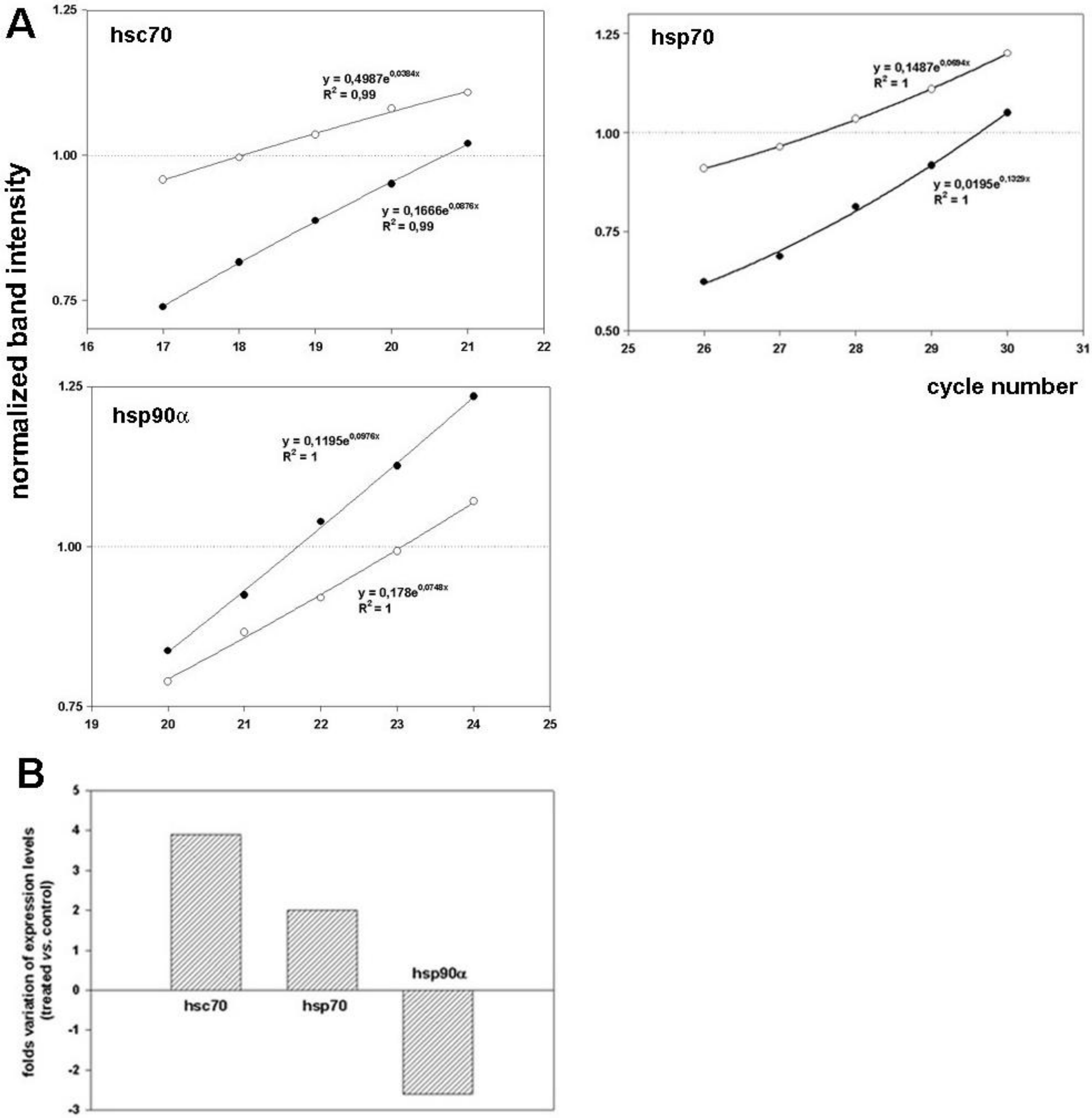

**FIGURE 4.** (A) SM-PCR for stress response genes *hsc70*, *hsp70*, and *hsp90α*. Representative plots of normalized data vs. cycle number fit with an exponential curve for control (●) and midregion PTHrP-treated (○) MDA-MB231 cells. (B) Histogram showing changes of gene expression levels in treated MDA-MB231 cells vs. controls, as resulted from SM-PCR assays.

equivalent amounts of proteins from triplicate samples were submitted to SDS-PAGE in a sequencing gel apparatus and to silver stain, according to Luparello[69], in order to reveal the expanded monodimensional protein patterns in both experimental conditions of culture. Interestingly, as shown in Fig. 5, although starting with equivalent amounts of proteins, preparations from treated cells displayed a noticeable weakening of the staining of all electrophoretic bands with respect to control samples, except for two protein bands of apparent $M_r$ of about 38 and 64 kDa, whose characterization remains to be determined. This result can be tentatively interpreted as a consequence of the augmentation of protein

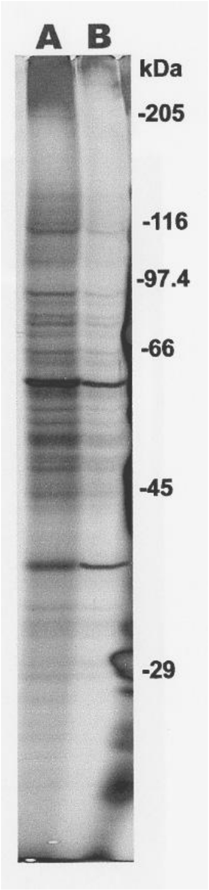

**FIGURE 5.** Electrophoretic analysis of monodimensional protein pattern from lysates of control (A) and PTHrP-treated (B) MDA-MB231 cell preparations. 10% SDS-PAGE, silver stain.

degradation events in PTHrP-treated cells, leading to loss of small protein fragments during the electrophoretic run. As discussed before, a role in this "protein loss" might be potentially ascribed to the up-regulation of *hsc70*; in fact, involvement of its protein product in chaperone-mediated autophagy, with an hsc70-induced increase of substrate uptake by the lysosomes and subsequent degradation, has been described[70]. This hypothesis is also supported by the absence of oligonucleosomal degradation of DNA in PTHrP-treated MDA-MB231 cells, as elsewhere reported. However, additional experiments are required to assess if PTHrP (38-94) is an actual stimulator of intracellular proteolytic activity, and which is the mechanism switched on by incubation of breast cancer cells with 1-nM midregion PTHrP.

## CONCLUSIONS

In the present review, more updated data on PTHrP (38-94)–mediated regulation of the breast cancer cell phenotype, obtained on the MDA-MB231 cell line chosen as the model system, have been summarized. Although future work is needed to define the precise nature of molecular events underlying PTHrP cytotoxicity and to expand the list of neoplastic cytotypes tested, the data so far obtained allow the following considerations.

Clear indications have been produced that PTHrP (38-94)-amide gains access inside MDA-MB231 cells and is imported into the nucleus where it is likely to bind chromatin in accordance with the ability demonstrated *in vitro*. Parallel data strongly suggest that the peptide actively controls gene expression, since exposition of cells to midregion PTHrP induces modifications in the expression level of different genes, including some of those encoding for stress response and apoptotic factors. Thus, new sets of experiments will be designed with the aim to supplement even further the catalogue of midregion PTHrP-responsive genes in MDA-MB231 cells, in order to better characterize the molecular basis of the putative "anti–breast cancer" effect of the peptide; on the other hand, to check its transcriptional factor–like role, it will be of interest to perform reporter gene studies to confirm *in vivo* the effect of midregion PTHrP on the modulation of gene expression.

From the preliminary data obtained, the lethal effect exerted by the peptide shows aspects amenable to both apoptosis and necrosis type of death; it is therefore conceivable that midregion PTHrP activates one of those alternative pathways of apoptosis, such as necrosis-like programmed cell death, which display mixed characteristics[e.g., 71]. Within this context, a particular interest will be devoted to the observed up-regulation of *Rip-1* and its biological significance; in fact, its protein product, whose key role in life/death cellular decisions has been cited before, was also proven to have properties of both apoptosis and necrosis factor. In particular, Holler et al.[72] reported that activation of Rip-1 kinase was responsible for the onset of cell necrosis due to accumulation of reactive oxygen species (ROS); moreover, Lewis et al.[73] and Vanden Berghe et al.[74] also demonstrated that hsp90, whose underexpression was observed in the model system for the $\alpha$ isotype, associates with Rip-1, thereby regulating its stability and activity. Thus, future investigation will be designed to check whether (1) mitochondrial respiration levels and intracellular redox state may be involved in PTHrP-mediated lethal effect and (2) hsp90$\alpha$ may play a role in the Rip-1–controlled cellular events in the model system. Lastly, the activation of extensive intracellular proteolysis by incubation with the peptide will be evaluated more in detail and the involved enzymes identified.

In conclusion, these studies provide evidence that PTHrP, in addition to its ability to activate endocrine, paracrine, and autocrine receptor-mediated processes, also likely possesses an intrinsic DNA binding and transcriptional modulation capability, which would appear to account, at least in part, for the well-documented nuclear localization of PTHrP and for the altered function of NTS-deleted forms of PTHrP. Moreover, these studies further support the concept that the physiology of PTHrP in normal breast development and function might include forms of PTHrP beyond those that have been studied most extensively to date, i.e., PTHrP (1-36) and full-length PTHrP[e.g., 12,21]. Based on the current observations, midregion PTHrP may seemingly play physiologic roles in mammary ductular development, fat pad invasion, branching morphogenesis, and/or lactation. Characterization of these possibilities will require further analysis.

## ACKNOWLEDGMENTS

This work was supported by a grant from the University of Palermo (R.S. ex 60%). We thank Prof. A.F. Stewart (University of Pittsburgh) for the generous gift of purified PTHrP (38-94)-amide and for helpful suggestions.
